# Neoadjuvant Chemoimmunotherapy in Non-small-cell Lung Cancer Patients: Effects on Pulmonary Function and Incidence of Postoperative Pulmonary Complications—A Multicentre Real-World Study

**DOI:** 10.1093/ejcts/ezaf351

**Published:** 2025-10-16

**Authors:** Jian Zhang, Yi Feng, Shaohua Dai, Guoqiu Xu, Bo Cheng, Lei Jiang, Li Peng Xue, Wenhua Liang, Jian Tang

**Affiliations:** Department of Thoracic Surgery, Jiangxi Clinical Research Center for Respiratory Diseases, The First Affiliated Hospital, Jiangxi Medical College, Nanchang University, Nanchang 330006, China; Department of Thoracic Oncology and Surgery, Guangzhou Institute of Respiratory Health, China State Key Laboratory of Respiratory Disease, National Clinical Research Center for Respiratory Disease, The First Affiliated Hospital, Guangzhou Medical University, Guangzhou 510120, China; Department of Thoracic Surgery, Jiangxi Clinical Research Center for Respiratory Diseases, The First Affiliated Hospital, Jiangxi Medical College, Nanchang University, Nanchang 330006, China; Department of Thoracic Surgery, Jiangxi Clinical Research Center for Respiratory Diseases, The First Affiliated Hospital, Jiangxi Medical College, Nanchang University, Nanchang 330006, China; Department of Thoracic Oncology and Surgery, Guangzhou Institute of Respiratory Health, China State Key Laboratory of Respiratory Disease, National Clinical Research Center for Respiratory Disease, The First Affiliated Hospital, Guangzhou Medical University, Guangzhou 510120, China; Department of Thoracic Surgery, Jiangxi Clinical Research Center for Respiratory Diseases, The First Affiliated Hospital, Jiangxi Medical College, Nanchang University, Nanchang 330006, China; Department of Thoracic Oncology and Surgery, Guangzhou Institute of Respiratory Health, China State Key Laboratory of Respiratory Disease, National Clinical Research Center for Respiratory Disease, The First Affiliated Hospital, Guangzhou Medical University, Guangzhou 510120, China; Department of Thoracic Oncology and Surgery, Guangzhou Institute of Respiratory Health, China State Key Laboratory of Respiratory Disease, National Clinical Research Center for Respiratory Disease, The First Affiliated Hospital, Guangzhou Medical University, Guangzhou 510120, China; Department of Thoracic Surgery, Southern Hospital, Southern Medical University, Guangzhou 510515, China

**Keywords:** neoadjuvant chemoimmunotherapy, pulmonary function tests, postoperative pulmonary complications

## Abstract

**Objectives:**

To evaluate the effects of neoadjuvant chemoimmunotherapy on pulmonary function and the incidence of postoperative pulmonary complications in real-world non-small-cell lung cancer patients.

**Methods:**

A retrospective analysis of stage II-IIIB non-small-cell lung cancer patients who received neoadjuvant chemoimmunotherapy across 3 medical institutions was conducted. Clinical data and perioperative outcomes were evaluated.

**Results:**

A total of 386 patients were screened and enrolled in the study cohort, and all patients underwent surgery after completing neoadjuvant chemoimmunotherapy. Postoperatively, 61 patients developed postoperative pulmonary complications, among whom 25 were diagnosed with checkpoint inhibitor-related pneumonia. The postoperative mortality rate was 2.8% (11/386), with all deaths attributed to severe postoperative checkpoint inhibitor-related pneumonia. The diffusing capacity of the lung for carbon monoxide tended to decrease after neoadjuvant chemoimmunotherapy, with 39% of patients developing impaired pulmonary function post-treatment. After controlling for confounding variables via propensity score matching analysis, impaired pulmonary function after treatment was associated with postoperative pulmonary complications. Multivariable logistic regression analysis revealed that prior lung disease (odds ratio, [OR]: 1.63; 95% CI, 1.02-3.31, *P* = .037), impaired pulmonary function after treatment (OR: 2.78; 95% CI, 1.38-5.24, *P* < .001), lymph node metastasis (OR: 3.05; 95% CI, 1.65-6.21, *P* < .001), and elevated preoperative interleukin-6 levels (OR: 1.58; 95% CI, 1.36-1.83, *P* < .001) were high risk factors for postoperative pulmonary complications.

**Conclusions:**

Impaired pulmonary function after neoadjuvant chemoimmunotherapy is associated with the development of postoperative pulmonary complications. Post-treatment impaired pulmonary function, lymph node metastasis, prior lung disease, and elevated preoperative interleukin-6 levels are risk factors for postoperative pulmonary complications.

**Clinical Registration Number:**

A Retrospective Study on Pulmonary Function Changes and Safety in Patients Receiving Neoadjuvant Chemoimmunotherapy (IIT2024733).

## Graphical abstract

**Figure:**
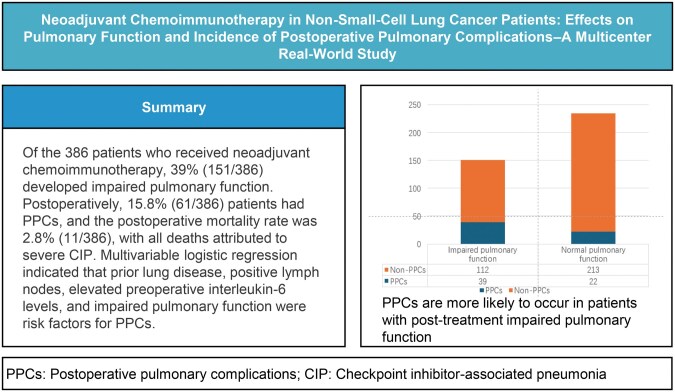

## INTRODUCTION

Neoadjuvant chemoimmunotherapy has emerged as a novel treatment modality over the past decade, demonstrating superior antitumour efficacy compared to neoadjuvant chemotherapy alone while promoting early immune memory development for enhanced long-term protection.^1^ Multiple clinical trials have confirmed improved prognoses in patients receiving neoadjuvant chemoimmunotherapy.^2–4^ Following the publication of these trial results and their incorporation into treatment guidelines, neoadjuvant chemoimmunotherapy has been widely adopted for stage II-IIIB non-small-cell lung cancer (NSCLC) patients.^5^ Despite the favourable safety profile reported in clinical trials, real-world data remain limited. With respect to the mechanism of neoadjuvant therapy, most neoadjuvant treatment modalities may potentially affect normal lung tissue to varying degrees, leading to pulmonary function changes that may influence postoperative complications rates.^6^ Previous studies exploring the relationship between pulmonary function changes and postoperative complications have primarily focused on radiotherapy or chemoradiotherapy, with limited specific data on pulmonary function changes and postoperative complications in patients receiving neoadjuvant chemoimmunotherapy.^7–9^ Real-world clinical settings present more complex and variables scenarios than randomized trials due to less stringent patient selection criteria. Given the current importance of the neoadjuvant chemoimmunotherapy in lung cancer treatment, disclosing safety outcomes in real-world settings is crucial for identifying potential challenges and risks. Therefore, this study aims to evaluate pulmonary function changes and the incidence of postoperative pulmonary complications (PPCs) in patients receiving neoadjuvant chemoimmunotherapy, investigate their potential association, and identify high-risk factors for PPCs.

## METHODS

### Ethical statement

This study obtained approval from the ethics committees of all participating centres. As a retrospective study requiring no additional interventions, patient informed consent was waived (IIT2024733, Approved 11/18/2024).

### Patients

**Figure 1.**
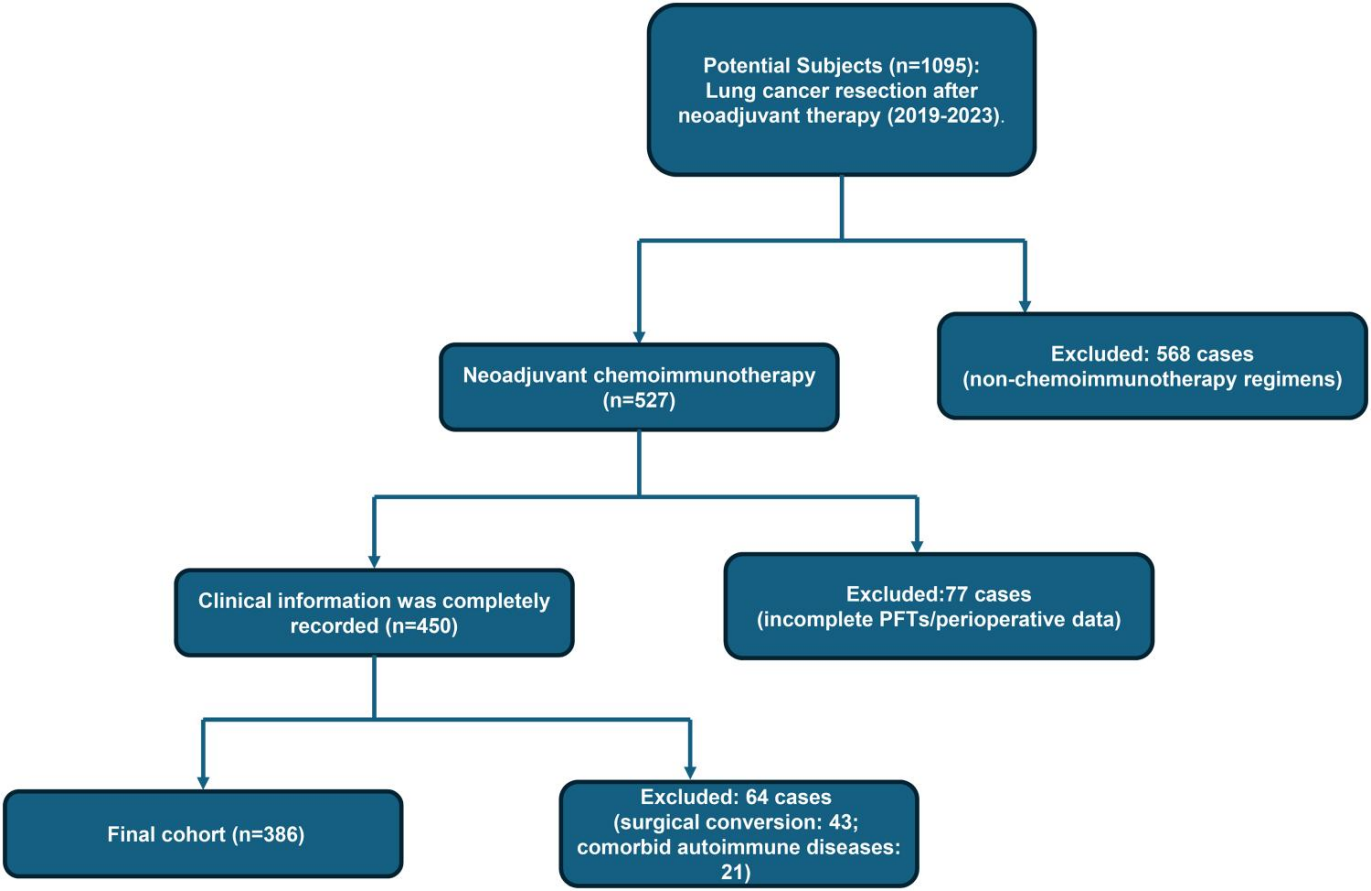
Participant Enrollment and Patient Screening Flow Diagram According to CONSORT. Abbreviation: PFT: pulmonary function test

The study followed the Strengthening the Reporting of Observational Studies in Epidemiology (STROBE) reporting guidelines for cohort studies. We retrospectively reviewed 1163 patients treated with neoadjuvant therapy. Sixty-eight patients were excluded from surgery due to disease progression, altered treatment intent, or an unfavourable preoperative assessment. All patients underwent preoperative multidisciplinary evaluation using a guideline recommended risk stratification system (see **Supplementary Material**). Finally, 1095 patients subsequently proceeded to surgery. Among these patients, 568 were excluded because their neoadjuvant regimen did not include chemoimmunotherapy (ie, immunotherapy, chemoradiotherapy, chemotherapy, or targeted agents); 77 were excluded because of a lack of data; 43 were excluded due to unplanned conversion to thoracotomy; and 21 were excluded because of comorbid autoimmune diseases. Ultimately, 386 patients were included in the final cohort (**Figure 1**).

### Assessment of study end-points and key variables

#### Pulmonary function tests and impaired pulmonary function

All patients underwent pulmonary function tests (PFTs) including forced expiratory volume in 1 second (FEV1), forced vital capacity (FVC), and diffusing capacity of the lung for carbon monoxide (DLCO) measurements before and after neoadjuvant chemoimmunotherapy. DLCO values were haemoglobin-corrected to avoid anemia related errors. Considering the primary difference in DLCO between pre- and post-neoadjuvant therapy in our cohort, and the potential of chemoimmunotherapy to induce lung interstitial injury, this study followed interstitial lung disease guidelines and defined post-treatment DLCO decline ≥15% as impaired pulmonary function.^10^

#### Postoperative pulmonary complications

The study primary end-point comprised respiratory system-related complications occurring within 30 days after surgery and grade ≥3 according to the Clavien-Dindo Classification of Surgical Complications,^11^ including checkpoint inhibitor-related pneumonia (CIP) and other non-CIP PPCs. All complications were prospectively documented, with all patients receiving enhanced recovery after surgery (ERAS). The diagnostic criteria for PPCs were based on the international multidisciplinary classification criteria for interstitial pneumonia by the American Thoracic Society/European Respiratory Society and guidelines for perioperative clinical outcomes issued by the European Joint Task Force.^12^^,^^13^

#### Statistical analysis

We employed propensity score matching (PSM) to balance confounding factors and clarify the association between impaired pulmonary function and PPCs. Nearest neighbour matching was used with a calliper set at 0.1 times the standard deviation of the logit-transformed propensity scores, and a 1:1 matching ratio between the impaired pulmonary function group and the normal pulmonary function group. Baseline characteristics were compared using t-tests for continuous variables, chi-square tests for categorical variables, and Fisher’s exact test for small-sample categorical variables. Multivariable logistic regression identified associations between PPCs and clinical characteristics, with variable selection based on least absolute shrinkage and selection operator (LASSO) regression and clinical relevance using enter stepwise procedure. Results are presented as odds ratio (OR), 95% confidence intervals (CIs), and *P* values. All the statistical analyses were performed using R version 4.4.3.

## RESULTS

### Clinical characteristics and treatment outcomes

Among the 386 patients, the mean age was 62 (56-69) years, and 70.2% (271/386) were male. Diabetes was the most common comorbidity, and chronic obstructive pulmonary disease (COPD) predominated among those with prior lung disease. All patients underwent programmed death ligand 1 (PD-L1) level testing before treatment, with 59.6% (230/386) having PD-L1 levels ranging from 1 to 49. A total of 66 (17.1%) patients experienced adverse events (grade ≥2) during treatment. One hundred eight patients achieved major pathological response (MPR), and 84 patients achieved pathological complete response (pCR) (**Table 1**). MPR and pCR rates showed no significant differences across age, gender, or PD-L1 expression levels. When stratified by tumour stage, pCR rates were significantly higher in stage IIA (45.71%) and IIB (28.81%) patients than in stage IIIA (13.51%) and IIIB (24.30%) patients (**Table S1**).

**Table 1.** Clinical Characteristics and Treatment Outcomes of Patients

Characteristic	*n* (%) or median (IQR)
Age (years)	62 (56-69)
Male	271 (70.2)
Female	115 (29.8)
BMI (kg/m²)	23.5 (21.3-25.5)
Smoking history	
Never	183 (47.4)
Ever	203 (52.6)
Comorbidity	
Hypertension	113 (29.3)
Diabetes	135 (35.0)
Coronary heart disease	75 (19.4)
Heart failure	82 (21.2)
Arrhythmiaª	13 (3.3)
Renal insufficiency	46 (11.9)
Prior lung disease	
COPD	96 (24.9)
Bronchiectasis	21 (5.4)
Asthma	14 (3.6)
Interstitial lung disease	4 (1.0)
Post-tuberculous sequelae	8 (2.1)
PD-L1 expression level	
<1	53 (13.7)
1-49	230 (59.6)
≥50	103 (26.7)
Clinical stage	
IIA	35 (9.07)
IIB	59 (15.28)
IIIA	185 (47.93)
IIIB	107 (27.72)
Adverse events during therapy	66 (17.1)
Neoadjuvant therapy cycles	3.0 (2.0-4.0)
Time interval (days)°	39.0 (31.0-48.0)
Degree of pathological response	
MPR	108 (28.0)
pCR	84 (21.8)

ª Includes patients diagnosed with cardiac arrhythmia via 24-hour Holter monitoring; °refers to the number of days between the completion of neoadjuvant chemoimmunotherapy and surgery in patients.

Abbreviations: BMI: body mass index; COPD: chronic obstructive pulmonary disease; MPR: major pathological response; pCR: pathological complete response; PD-L1: programmed death-ligand 1.

### Changes in pulmonary function and perioperative characteristics

**Figure 2.**
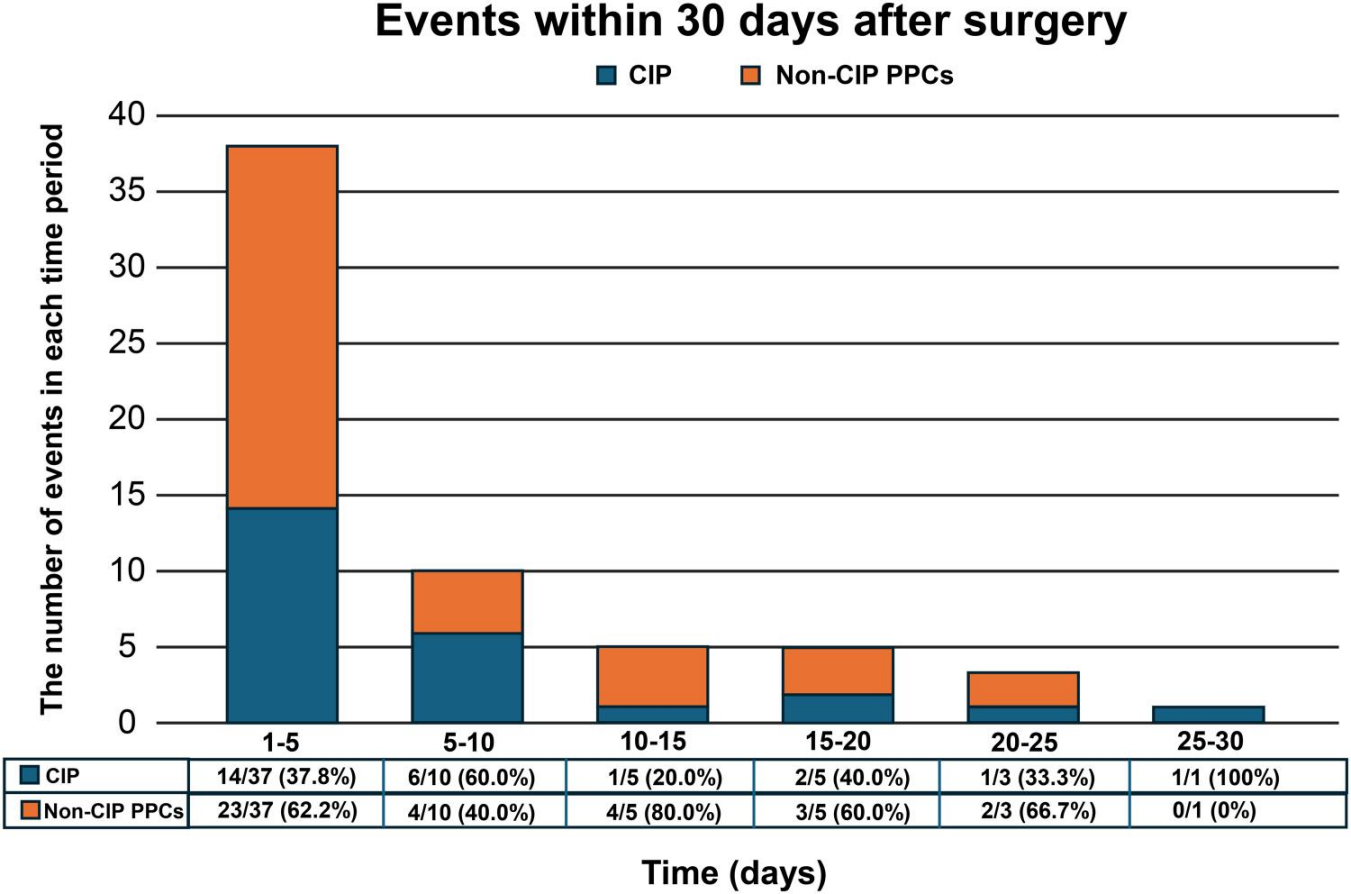
Temporal Distribution of Postoperative Pulmonary Complications Within 30 Days: Checkpoint Inhibitor-related Pneumonia (CIP) vs Non-CIP Postoperative Pulmonary Complications. Abbreviation: PPC: postoperative pulmonary complication

The median pretreatment FEV1 was 90% (IQR, 80.7%-97.6%), median pretreatment FVC was 95.8% (88.0%-102.3%), and median pretreatment DLCO was 81.0% (75.0%-86.0%). Median post-treatment FEV1 was 89.4% (81.0%-96.2%), median post-treatment FVC was 94.9% (87.1%-103.5%), and median post-treatment DLCO was 68.0% (61.4%-77.4%). Pulmonary function changes primarily affected DLCO, which decreased significantly after neoadjuvant therapy; 151 (39.1%) patients developed impaired pulmonary function. Surgical procedures included video-assisted thoracic surgery (VATS) in 279 patients (72.28%) and open thoracotomy in 90 patients (23.32%). Lobectomy was the predominant resection type (337, 83.81%), with 6 patients undergoing extended lobectomy. Mean operative time was 148.33 ± 44.66 min, with mean blood loss of 149.32 ± 58.16 mL. The mean number of lymph node dissection stations was 6.95 ± 1.25. A total of 61 patients (15.80%) developed PPCs, among whom 25 (6.48%) were diagnosed with checkpoint inhibitor-related pneumonia (CIP). Other PPCs included infectious pneumonia (14 cases), hypoxemia (9 cases), bronchospasm (3 cases), and acute respiratory failure (10 cases). The number of deaths was 11, all attributed to severe postoperative CIP. Compared to patients with normal pulmonary function, those in the impaired PFTs group had significantly longer intensive care unit (ICU) length of stay, longer mean total postoperative hospital stay, and greater number of patients with PPCs (**Table 2**). The distribution of PPCs across different time intervals is shown in **Figure 2**. Events were predominantly concentrated within the first 10 days postoperatively, with the highest number of cases occurring on days 1-5 postoperatively for PPCs (**Figure 2**).

**Table 2.** Comparison of Perioperative Data and Postoperative Outcome in Patients with Different Pulmonary Function Status After Treatment

Characteristic	Total (*n* = 386)	Impaired PFTs group (151)	Normal PFTs group (235)	Statistic	*P*
Surgical methods				χ²=0.00	.974
VATS	279 (72.28%)	109 (72.19%)	170 (72.34%)		
Open thoracotomy	107 (27.72%)	42 (27.81%)	65 (27.66%)		
Extent of resection				–	.874
Lobectomy	337 (87.31%)	134 (88.74%)	203 (86.38%)		
Bilobectomy	32 (8.29%)	12 (7.95%)	20 (8.51%)		
Sleeve resection	11 (2.85%)	3 (1.99%)	8 (3.40%)		
Extended lobectomy	6 (1.55%)	2 (1.32%)	4 (1.70%)		
Operative time (min)	148.33 ± 44.66	150.89 ± 47.07	146.68 ± 43.05	t = 0.91	.366
Intraoperative blood lose (mL)	149.32 ± 58.16	151.03 ± 59.70	148.23 ± 57.26	t = 0.46	.645
Number of lymph nodes resected	16.12 ± 8.37	15.79 ± 8.00	16.34 ± 8.61	t = −0.63	.531
Number of lymph node dissection station	6.95 ± 1.25	7.08 ± 1.23	6.87 ± 1.26	t = 1.59	.112
ICU length of stay (days)	2.67 ± 2.21	3.26 ± 3.23	2.29 ± 1.00	t = 3.58	<.001
Postoperative hospital length of stay	8.22 ± 4.52	9.42 ± 5.77	7.45 ± 3.27	t = 3.81	<.001
Postoperative CIP	25 (6.48%)	21 (13.91%)	4 (1.7%)	χ² = 20.94	<.001
PPCs	61 (15.80%)	39 (25.83%)	22 (9.36%)	χ² = 29.68	<.001
Deaths	11 (2.84%)	8 (5.30%)	3 (1.27%)	χ² = 4.10	.043

Impaired PFTs group: patients with a DLCO decline of ≥15% after neoadjuvant chemoimmunotherapy are defined as the impaired PFTs group; postoperative pulmonary complications include postoperative CIP, and other postoperative pulmonary complications include pneumonia (14), hypoxemia (9), bronchospasm (3), and acute respiratory failure (10); t: t-test; χ²: chi-square test; -: Fisher exact.

Abbreviations: CIP: checkpoint inhibitor-related pneumonia; PPCs: postoperative pulmonary complications; VATS: video-assisted thoracic surgery.

### Relationship between impaired pulmonary function and PPCs

In this retrospective cohort study, we performed PSM to reduce the impact of selection bias and confounding factors and to demonstrate the potential association between impaired pulmonary function and PPCs. The covariates included in the PSM model were age, gender, smoking history, prior lung disease, PD-L1 expression level, and the adverse events during treatment. Before matching, significant differences existed between the 2 groups in terms of gender, smoking history, and incidence of adverse events during treatment. After matching, the standardized mean difference (SMD) for all confounding factors was <10%, indicating that balance was achieved (**Table S2**). PPCs occurred in 27.21% of the impaired PFTs group compared with 7.35% of the normal PFTs group (*P* < .001, χ^2^=18.75) (**Table S3**).

### Multivariable logistic regression analysis of risk factors for PPCs

Considering the limited number of target events, we performed variable selection using LASSO regression and clinical considerations. The variables selected by LASSO regression included age, smoking history, prior pulmonary disease, adverse events during neoadjuvant chemoimmunotherapy, post-treatment impaired pulmonary function, lymph node metastasis, and preoperative interleukin-6 (IL-6) elevation. Variables selected from the clinical perspective were extent of excision and PD-L1 expression level. The results of the multivariable logistic regression model revealed that prior lung disease (OR: 1.63; [95% CI, 1.02-3.31], *P* = .037), impaired pulmonary function (OR: 2.78; [95% CI, 1.38-5.24], *P* < .001), lymph node metastasis (OR: 3.05; [95% CI, 1.65-6.21], *P* < .001), and preoperative IL-6 elevation (OR: 1.58; [95% CI, 1.36-1.83], *P* < .001) were significantly associated with the occurrence of PPCs (**Table 3**).

**Table 3.** Multivariable Regression to Explore Risk Factors for PPCs

Variables	Univariable analysis			Multivariable analysis		
	β	*P*	OR (95%CI)	β	*P*	OR (95%CI)
Age	−0.01	.407	0.99 (0.96-1.02)	−0.02	.404	0.98 (0.95-1.02)
Smoking history						
Never			1.00 (Reference)			1.00 (Reference)
Ever	0.55	.055	1.74 (0.99-3.07)	0.32	.378	1.38 (0.68-2.80)
Prior lung disease	0.72	.03	1.92 (1.17-3.95)	0.59	.037	1.63 (1.02-3.31)
PD-L1						
<1			1.00 (Reference)			1.00 (Reference)
1-49	−0.79	.237	0.45 (0.23-0.91)	−0.56	.223	0.57 (0.23-1.41)
≥50	−1.19	.117	0.30 (0.13-0.72)	−0.99	.081	0.37 (0.12-1.13)
Adverse events during therapy	0.45	.188	1.56 (0.80-3.05)	1.68	.315	1.27 (0.95-4.11)
Extent of excision						
Bilobectomy			1.00 (Reference)			1.00 (Reference)
Extended lobectomy	−15.1	.988	0.00 (0.00-Inf)	−14.37	.988	0.00 (0.00-Inf)
Lobectomy	−0.19	.69	0.83 (0.32-2.10)	−0.45	.437	0.64 (0.21-1.98)
Sleeve resection	−0.84	.464	0.43 (0.05-4.07)	−1.25	.366	0.29 (0.02-4.32)
Impaired pulmonary function	1.49	<.001	4.44 (2.46-7.13)	1.02	<.001	2.78 (1.38-5.24)
Lymph node metastasis	1.62	<.001	4.08 (2.25-7.40)	1.27	<.001	3.05 (1.65-6.21)
Preoperative IL-6 elevation	0.25	<.001	1.29 (1.20-1.39)	0.46	<.001	1.58 (1.36-1.83)

Inf: The data distribution of surgical resection scope shows individual extremization; included variables were derived from LASSO regression and clinically relevant considerations; LASSO regressions selected the appropriate λ based on cross-validation results. For multivariable logistic regressions, no *P* value threshold was set for included variables, and all were included using the Enter method.

Abbreviations: CI: confidence interval; OR: odds ratio.

## DISCUSSION

The safety profile of neoadjuvant chemoimmunotherapy in real-world practice requires further elucidation. While clinical trials report favourable outcomes, the inherent differences between real-world scenarios and randomized trials necessitate a dedicated evaluation of this treatment’s impact on pulmonary function and postoperative complications. In our retrospective analysis of 386 patients, we observed that 151 patients developing impaired pulmonary function after treatment, and 61 patients developed PPCs. Furthermore, post-treatment impaired pulmonary function, prior lung disease, lymph node metastasis, and elevated preoperative IL-6 levels were risk factors for PPCs.

Although our complication rates are lower than those reported in prior studies (15.8%vs.18.9%/21%),^14^^,^^15^ directed comparisons are not appropriate due to fundamental differences in study design and patient populations. Previous research predominantly involved randomized controlled trials (RCTs) that establish highly homogeneous patient cohorts through strict inclusion and exclusion criteria. These patients typically present with lower comorbidity burdens and better physical function, alongside ideal treatment adherence, monitoring completeness, and follow-up standardization under trial management. In contrast, real-world clinical practice encompasses highly heterogeneous patient populations with more complex comorbidities, factors often excluded from RCTs that likely significantly impact perioperative safety. Furthermore, definitions of postoperative safety events differ: RCTs use broad criteria to fully capture treatment-related adverse events, often including common post-lung-surgery issues like pleural effusion and atelectasis, while our study focuses on postoperative complications from neoadjuvant chemoimmunotherapy-related specific pulmonary function damage to better reflect the treatment’s inherent safety problems. Thus, although our complication incidence appears lower than RCTs reports, this finding should not be considered optimistic given that the study end-point was a specific type of complication. As neoadjuvant chemoimmunotherapy application expands, the safety of this treatment in real-world populations warrants further investigation.

Our analysis revealed that neoadjuvant chemoimmunotherapy predominantly affects DLCO, with minimal impact on FEV1 or FVC. This phenomenon is consistent with previous studies.^8^^,^^9^ DLCO reflects the gas exchange function of the alveolar-capillary membrane, and its decline directly indicates structural damage to the alveolar epithelium, capillary endothelium, or lung interstitium.^16^ This damage potentially mediated by chemotherapeutic pneumotoxicity combined with immune checkpoint inhibitor-induced aberrant immune responses.^17–19^ Unlike the ventilatory function represented by FEV1 and FVC, DLCO is more difficult to reverse through respiratory rehabilitation exercises. Therefore, in patients receiving neoadjuvant therapy, declines in pulmonary function are more likely to be reflected in DLCO.^8^^,^^9^^,^^20^^,^^21^

Our identification of post-treatment impaired pulmonary function, prior lung disease, lymph node metastasis, and elevated preoperative IL-6 levels as risk factors for PPCs shares similarities with existing literature. Cerfolio et al^9^ similarly reported that pulmonary function decline after neoadjuvant therapy predicts postoperative complications. Connolly JG et al also demonstrated that patients with impaired pulmonary function after treatment had a greater probability of developing complications.^22^ Our results revalidate this phenomenon, though with a nuanced difference in defining impaired pulmonary function. For example, in the study by Connolly JG et al, the assessment of impaired pulmonary function was based on a specific time point, that is, a preoperative DLCO value below a certain threshold was considered impaired pulmonary function,^22^ whereas our definition of impaired pulmonary function focuses on a dynamic change process: even if DLCO is within the normal range after treatment, a decline of ≥15% compared with pretreatment levels is considered impaired pulmonary function. Evaluating which definition is more effective is difficult, but it is certain that DLCO is a key parameter that clinicians should focus on in patients receiving neoadjuvant chemoimmunotherapy. Although not presented in the results section, additional analysis specifically examining postoperative CIP revealed that impaired pulmonary function, lymph node metastasis, and elevated preoperative IL-6 levels remained significantly associated. We hypothesize that the association with lymph node metastasis might stem from the possibility that immunotherapy could induce a long-term immunological response in patients. Lymph node metastases represent reservoirs of tumour antigens, and their surgical dissection may expose these antigens to an already activated immune system. Combined with surgical stress and postoperative mechanical ventilation, this could precipitate immune hyperactivation, increasing PPCs or CIP incidence. Sukhbaatar et al^23^ demonstrated in mouse models that tumour antigens release following lymph node resection, coupled with surgical trauma, activates the immune microenvironment, leading to increased infiltration of CD8+ T cells and natural killer cells. This finding offers a possible mechanistic basis for the phenomenon observed in our study. Elevated preoperative IL-6 levels may indicate that the patient’s immune-system is already activated before surgery, making such patients more prone to PPCs or CIP.

Multivariable logistic regression identified prior lung diseases as a PPC risk factor; however, we propose considering a potential mediating effect. Studies have shown that patients with such diseases are more likely to develop DLCO impairment during neoadjuvant therapy, suggesting that this factor may affect PPC development via DLCO impairment. Thus, when these clinical features are used to develop risk scores for screening high-risk patients, caution is needed to avoid overemphasizing either direct or mediating effects. Nonetheless, clinical attention to such patients remains important to ensure the safety of neoadjuvant chemoimmunotherapy.

Our study has several limitations. First, although this was a multicentre study, only 3 institutions were included as other institutions were limited by incomplete data. This may introduce certain biases into our study. We plan to design larger-scale multicentre retrospective studies and develop a predictive model for perioperative complications of neoadjuvant chemoimmunotherapy to address this issue. Second, we failed to definitively determine the optimal inflection point of DLCO decline for impaired pulmonary function, likely because of insufficient samples with severe DLCO reduction, which limits statistical power. Future studies with larger sample sizes are needed to clarify this risk inflection point. Third, the study did not perform survival analysis or quality-of-life analysis for patients with impaired pulmonary function after treatment, leaving unanswered whether post-treatment pulmonary function impairment affects prognosis or leads to decreased quality of life after surgery. We are currently collecting data in these areas and plan to present and discuss the results further in the future.

In conclusion, based on the collected data, we observed an association between impaired pulmonary function after neoadjuvant chemoimmunotherapy and PPCs and identified prior lung disease, post-treatment impaired pulmonary function, lymph node metastasis, and elevated preoperative IL-6 levels as high-risk factors for PPCs.

## Supplementary Material

ezaf351_Supplementary_Data

## Data Availability

The data underlying this article cannot be shared publicly due to the fact that the original data contain patient hospital numbers and basic information, and to protect patient privacy. The data will be shared on reasonable request to the corresponding author.
