# Supplementary material for: Neoadjuvant Chemoimmunotherapy in Non-small-cell Lung Cancer Patients: Effects on Pulmonary Function and Incidence of Postoperative Pulmonary Complications—A Multicentre Real-World Study
Source: Eur J Cardiothorac Surg. 2025 Oct 16;67(11):ezaf351. doi: 10.1093/ejcts/ezaf351 (PMC12597875; doi:10.1093/ejcts/ezaf351)
Supplement: ezaf351_Supplementary_Data [file ezaf351_supplementary_data.zip › Supplementary material.docx]

In the patient screening section of our manuscript, we only described the screening process starting from patients who underwent surgery after completing neoadjuvant therapy. During the peer review of this manuscript, we received suggestions from reviewers that we should clarify the specific population of patients who received neoadjuvant chemoimmunotherapy but ultimately did not undergo surgery. The reasons for dropout in this population may, to a certain extent, introduce bias into the study results. Therefore, we have supplemented the description of this part of the data and elaborated on the specific criteria for preoperative evaluation during the study. Considering the manuscript length constraints and the need for comprehensive description, we have submitted this content as supplementary material for easy reference by interested readers.

During the initial patient screening for this study, there were 1163 lung cancer patients who received neoadjuvant therapy, among whom 1095 underwent surgery after completing neoadjuvant therapy, and 68 did not. The reasons for not undergoing surgery included disease progression during neoadjuvant therapy (12 patients), severe adverse reactions during neoadjuvant therapy, patients’ refusal of subsequent treatment (6 patients), changes in patients’ treatment willingness and transfer to other medical centers (11 patients), and failure to pass the preoperative multidisciplinary team (MDT) evaluation (39 patients). Given that different preoperative evaluation criteria may alter the number of patients ultimately undergoing surgery, thereby potentially introducing bias into postoperative outcomes, we have fully described the specific process and details of preoperative evaluation for patients receiving neoadjuvant therapy.

All patients who received neoadjuvant therapy underwent an MDT evaluation led by the thoracic surgery department to determine suitability for subsequent surgery after completing the scheduled neoadjuvant therapy regimen. The MDT comprised specialists from the thoracic surgery, anesthesiology, Intensive Care Unit (ICU), rehabilitation, and radiology departments. The evaluation dimensions included general condition and comorbidities, pulmonary function assessment, cardiac function assessment, other organ function assessment, and radiological assessment and staging.

General condition and comorbidities were evaluated using the American Society of Anesthesiologists (ASA) classification and Eastern Cooperative Oncology Group (ECOG) performance status[1][2]. Pulmonary function assessment included baseline pulmonary function and predicted postoperative pulmonary function. Cardiac function assessment involved electrocardiography, echocardiography, and myocardial marker testing. Assessment of other organ functions covered liver and kidney function, electrolyte levels, and coagulation function indicators. Radiological assessment included the response of the primary tumor and lymph nodes to treatment, as well as the presence of newly developed metastatic lesions.

Regarding the specific thresholds and criteria for pulmonary function assessment, we adopted the risk stratification system recommended by international authoritative guidelines (American College of Chest Physicians/European Society of Thoracic Surgeons [ACCP/ESTS])[3][4], with core indicators including forced expiratory volume in 1 second (FEV1), diffusing capacity of the lung for carbon monoxide (DLCO), and their predicted postoperative values (predicted postoperative FEV1 [ppoFEV1], predicted postoperative DLCO [ppoDLCO]). Low-risk patients had all indicators with absolute values or percentages of predicted values > 80% of the predicted value. Medium-risk patients had both ppoFEV1 and ppoDLCO > 40%, or ppoDLCO between 30% and 40% with maximum oxygen consumption (VO2max) > 20 mL/kg/min. High-risk patients had ppoFEV1 or ppoDLCO < 30% of the predicted value, or VO2max < 10 mL/kg/min. Patients at low or medium risk could undergo further surgical treatment under meticulous perioperative management, while high-risk patients, with extremely high risks of mortality and severe complications, were not recommended for surgery and required consideration of non-surgical treatment options.

Given that this is a retrospective study, the preoperative evaluation criteria used at that time were consistent with the criteria for defining impaired pulmonary function in this study. To a large extent, the purpose of this study was to clarify the perioperative risks of patients who passed preoperative evaluation after neoadjuvant chemoimmunotherapy but had a decline in DLCO compared with baseline.

Reference:

1. Oken MM, Creech RH, Tormey DC, et al. Toxicity and response criteria of the Eastern Cooperative Oncology Group. *Am J Clin Oncol*. 1982;5(6):649-655.
2. Mayhew D, Mendonca V, Murthy BVS. A review of ASA physical status - historical perspectives and modern developments. *Anaesthesia*. 2019;74(3):373-379. doi:10.1111/anae.14569
3. Brunelli A, Kim AW, Berger KI, Addrizzo-Harris DJ. Physiologic evaluation of the patient with lung cancer being considered for resectional surgery: Diagnosis and management of lung cancer, 3rd ed: American College of Chest Physicians evidence-based clinical practice guidelines. *Chest*. 2013;143(5 Suppl):e166S-e190S. doi:10.1378/chest.12-2395
4. Brunelli A, Charloux A, Bolliger CT, et al. ERS/ESTS clinical guidelines on fitness for radical therapy in lung cancer patients (surgery and chemo-radiotherapy). *Eur Respir J*. 2009;34(1):17-41. doi:10.1183/09031936.00184308
